# The Hand of *Cercopithecoides williamsi* (Mammalia, Primates): Earliest Evidence for Thumb Reduction among Colobine Monkeys

**DOI:** 10.1371/journal.pone.0125030

**Published:** 2015-05-20

**Authors:** Stephen R. Frost, Christopher C. Gilbert, Kelsey D. Pugh, Emily H. Guthrie, Eric Delson

**Affiliations:** 1 Department of Anthropology, 1218 University of Oregon, Eugene, Oregon, 97403–1218, United States of America; 2 Department of Anthropology, Hunter College of the City University of New York, New York, New York, 10065, United States of America; 3 New York Consortium in Evolutionary Primatology, New York, New York, 10024, United States of America; 4 PhD Program in Anthropology, The Graduate Center, City University of New York, New York, New York, 10016, United States of America; 5 Human Subjects Division, University of Washington, Seattle, Washington, 98185, United States of America; 6 Department of Vertebrate Paleontology, American Museum of Natural History, New York, New York, 10024, United States of America; 7 Department of Anthropology, Lehman College, City University of New York, Bronx, New York, 10468, United States of America; Midwestern University & Arizona State University, UNITED STATES

## Abstract

Thumb reduction is among the most important features distinguishing the African and Asian colobines from each other and from other Old World monkeys. In this study we demonstrate that the partial skeleton KNM-ER 4420 from Koobi Fora, Kenya, dated to 1.9 Ma and assigned to the Plio-Pleistocene colobine species *Cercopithecoides williamsi*, shows marked reduction of its first metacarpal relative to the medial metacarpals. Thus, KNM-ER 4420 is the first documented occurrence of cercopithecid pollical reduction in the fossil record. In the size of its first metacarpal relative to the medial metacarpals, *C*. *williamsi* is similar to extant African colobines, but different from cercopithecines, extant Asian colobines and the Late Miocene colobines *Microcolobus* and *Mesopithecus*. This feature clearly links the genus *Cercopithecoides* with the extant African colobine clade and makes it the first definitive African colobine in the fossil record. The postcranial adaptations to terrestriality in *Cercopithecoides* are most likely secondary, while ancestral colobinans (and colobines) were arboreal. Finally, the absence of any evidence for pollical reduction in *Mesopithecus* implies either independent thumb reduction in African and Asian colobines or multiple colobine dispersal events out of Africa. Based on the available evidence, we consider the first scenario more likely.

## Introduction

Extant colobine monkeys (Order Primates, Subfamily Colobinae) can be divided into two major groups, usually recognized as subtribes: the African Colobina, including the genera *Colobus* (black and black-and-white colobus monkeys) and *Procolobus* (red and olive colobus monkeys), and the Asian Presbytina, including the genera *Presbytis*, *Semnopithecus*, *Trachypithecus* (leaf monkeys and langurs), *Nasalis*, *Simias*, *Pygathrix*, and *Rhinopithecus* (odd-nosed monkeys) [1–6]. Molecular evidence corroborates the division of extant Colobinae into these two subtribes and also suggests that they diverged approximately 10–12 Ma [7–12].

Although fossil colobines are common in the late Miocene through Plio-Pleistocene fossil record, their relationships to the modern subtribes are currently unclear. For example, the African fossil record of colobines is extensive and considerably more diverse than the extant colobinan radiation with at least six extinct genera (*Microcolobus*, *Rhinocolobus*, *Paracolobus*, *Libypithecus*, *Kuseracolobus*, and *Cercopithecoides*) [13]. However, despite several partial crania and even a few partial skeletons being known for a number of genera [14–23], the phylogenetic relationships of these extinct forms to each other and to the living forms are not well resolved [2,4,5,17,19,24,25].

At present, there are a number of hypotheses regarding the phylogenetic relationships of late Miocene-Pliocene African fossil colobines. Delson [2,4,17,24] hypothesized that all of the extinct African fossil genera are stem colobinans, with the possible exception of *Microcolobus*, and he further suggested that *Libypithecus* may be more closely related to the extant African genera than the other fossil forms. Nakatsukasa et al. [23] recently provided strong evidence that *Microcolobus* possessed an unreduced pollex, based on a partial skeleton with incomplete MC I, MC II and complete MC IV (KNM-NA 47916). They showed that MC I base area was within the cercopithecine range when compared to MC IV length. Therefore, their finding supports previous assessments that *Microcolobus* is most likely a stem colobine [2,26].

Leakey [19,25] hypothesized that *Cercopithecoides* was likely a stem colobine not specifically related to either modern clade, but rather to the (semi-)terrestrial extinct Eurasian genera *Mesopithecus* and *Dolichopithecus*. This assessment was based, at least in part, on the fact that the preserved postcranial elements of *Cercopithecoides williamsi* suggest a semi-terrestrial to terrestrial monkey, similar to *Mesopithecus* and *Dolichopithecus* [18,21,22]. In addition, Leakey hypothesized that *Rhinocolobus* may have affinities to *Libypithecus* and the extant Asian genus *Nasalis* [19]. Jablonski [5] placed *Rhinocolobus* within the African colobine clade, but considered *Cercopithecoides* and the other African fossil genera of unknown affinity. Most recently, Jablonski et al. [22] proposed that *Rhinocolobus* was closely related to *Nasalis* and *Cercopithecoides williamsi* was closely related to *Mesopithecus* and *Semnopithecus*.

One of the most distinctive features of extant colobines is the reduction of the pollex relative to its condition in cercopithecines, with significantly greater reduction in colobinans than presbytinans [4]. In fact, the subfamily Colobinae and genus name *Colobus* derive from the Greek word for “maimed” or “mutilated”, in reference to the highly reduced-to-absent external thumb. Manual elements, including thumbs, are rare in the colobine fossil record, with remains of only two genera described to date: *Mesopithecus* [27] and *Microcolobus* [23]. Neither of these appears to show much, if any, reduction of the pollical phalanges or first metacarpal [4,23,28]. Here we report the state of first metacarpal reduction in *Cercopithecoides williamsi* preserved in the partial skeleton KNM-ER 4420 from the Upper Burgi Member of the Koobi Fora Formation [19,22]. We demonstrate that KNM-ER 4420 represents the earliest known evidence for significant thumb reduction in the colobine fossil record and, as such, it has significant bearing on the affinities of *C*. *williamsi* and the evolution of thumb reduction and manual morphology within the Colobinae.

## Materials and Methods

No living animals were used in this study. This analysis was carried out on specimens housed at institutions listed in the acknowledgments. All regulations at these institutions were followed. Permits were only required for the National Museum of Kenya, Nairobi and the National Museum of Ethiopia, Addis Ababa; these were issued by the Kenyan National Commission for Science, Technology and Innovation and by the Ethiopia Authority for Research and Conservation of the Cultural Heritage respectively.

**Fig 1:**
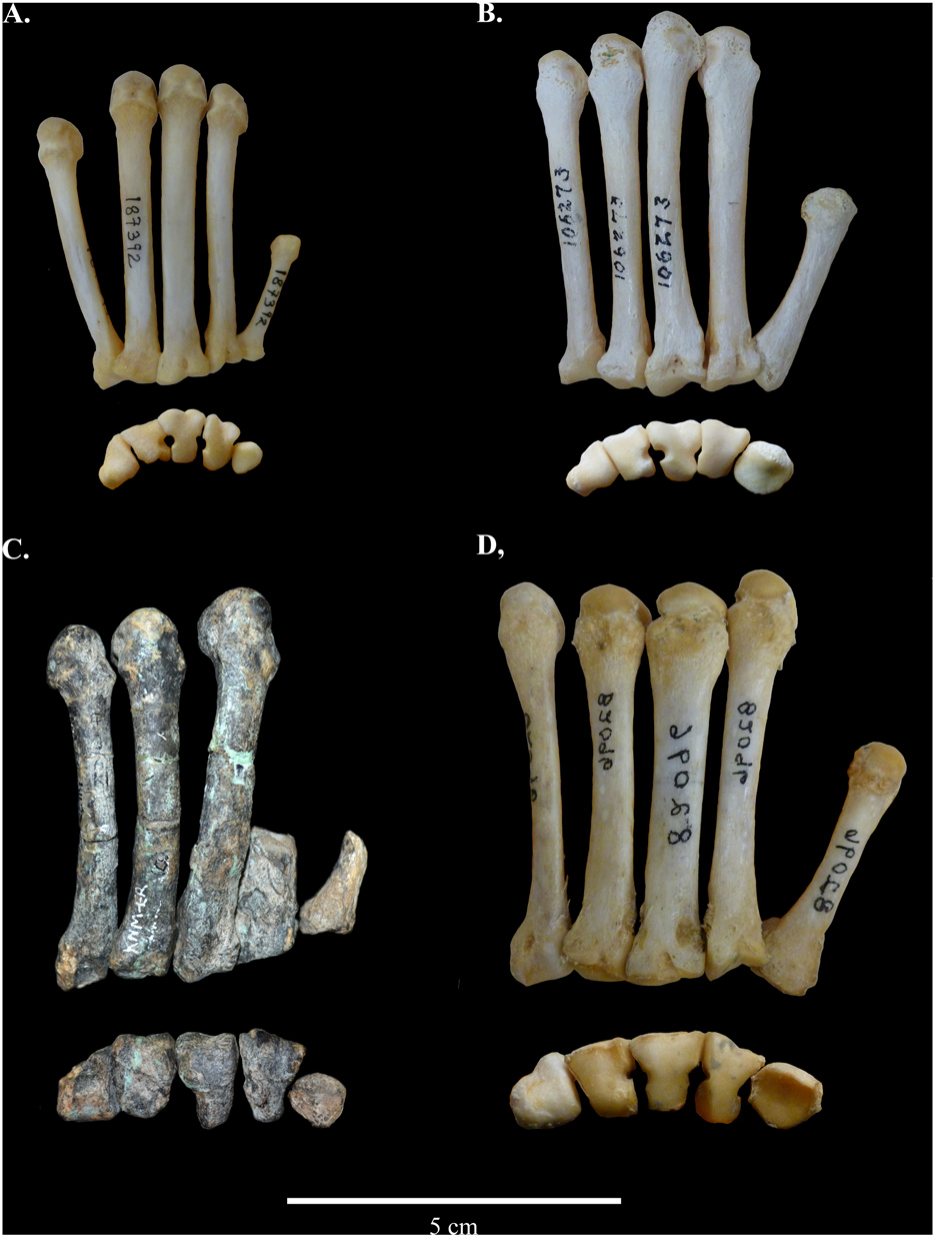
Left metacarpals of selected cercopithecids in approximate articulated position, dorsal and proximal views. A. *Colobus guereza*, female; B. *Nasalis larvatus* male; C. *Cercopithecoides williamsi* (KNM-ER 4420) male; D. *Papio hamadryas anubis* male (photographically reversed).

**Fig 2:**
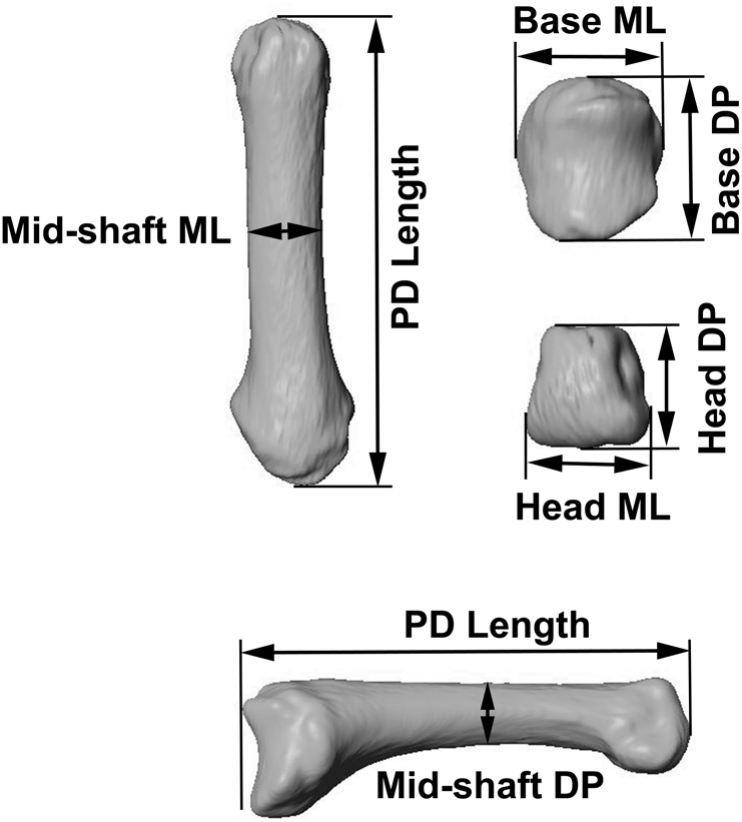
Metacarpal measurements presented in Table 1. For metric analyses, only proximodistal length, mediolateral breadth of the base, and dorsopalmar depth of the base were included.

KNM-ER 4420 includes portions of all five metacarpals on the left side. Metacarpal (MC) I preserves an intact base and what appears to be over half of the shaft. The distal end of MC I is not preserved. What is preserved of MC I suggests that the shaft would not have extended much further, due to the tapering already present. Overall, the shape of the preserved bone looks most similar in overall relative proportions to that seen in African colobines. MC II preserves an intact base and approximately one-third of the proximal shaft; the distal portion of MC II is also present but does not fit onto the proximal portion, due to some apparent distortion. Therefore, it is impossible to determine exactly how the proximal and distal portions of MC II relate to each other with any confidence or accuracy. MC III, MC IV, and MC V are all complete, with the exception of some damage to the MC V base and a few midshaft breaks that were repaired during preparation (Fig 1). The metacarpals of KNM-ER 4420 are typical of extant colobines, with a few notable exceptions in their morphology. First, they are noticeably larger, due to the large size of *Cercopithecoides williamsi* compared with living African colobines. Second, they are stouter and more robust in their proportions, as would be expected in a more terrestrial animal. Finally, also in line with a semi-terrestrial/terrestrial locomotor pattern, their shafts appear to be straighter than those of most living colobines, but more curved than is the case in papionins. Raw measurements on the MCs of KNM-ER 4420 are provided in Table 1 and illustrated in Fig 2.

**Table 1:** Standard measurements for left metacarpals of KNM-ER 4420.

Element	Length	Base DP	Base ML	Mid-shaft DP	Mid-shaft ML	Head DP	Head ML
**MC I**	-	8.3	7.7	-	-	-	-
**MC II**	-	12.9	9.6	6.2	5.9	11.6	9.4
**MC III**	58.5	13.3	9.2	5.9	7.2	10.8	9.7
**MC IV**	55.5	11.8	9.8	5.6	6.1	10.7	9.7
**MC V**	55.4	(9.9)	(8.9)	6.3	5.3	10.1	8.9

Notes: DP = dorso-palmar, ML = medio-lateral,— = unavailable measurement. Numbers in parentheses represent estimates. Measurements of MC II Mid-shaft and head are from separate distal MC II fragment.

For analysis, we examined a comparative sample consisting of 30 extant colobines and 41 extant cercopithecines (Table 2) as well as data from the literature on fossils of *Mesopithecus* (MNHN PIK 302 [27]) and *Microcolobus* (KNM-NA 47915 [23]). For each specimen in the comparative sample, three measurements were taken on all 5 metacarpals: the total proximo-distal bone length and the maximum medio-lateral width and dorso-palmar height of the base. All measurements were collected using digital calipers and recorded to the nearest tenth of a millimeter (Fig 2; S1 Table). All standard statistical analyses were performed in SPSS and PAST. In addition to standard statistics, phylogenetically corrected statistical analyses (phylogenetic least squares regressions and ANOVAS) were performed using the Caper package in R [29–30]. In phylogenetic least squares analyses, multiple specimens per species were treated as separate OTUs and enumerated with a number at the end of the species name (e.g., Colobus_guereza1, Colobus_guereza2, etc.). The corresponding tree treated the enumerated specimen names as polytomies to represent a single species. See S1 PGLSTree for the nexus file used in phylogenetic correction. For all regression analyses and ANOVAs both standard and phylogenetically corrected results are presented.

**Table 2:** Sample size for specimens included in this analysis. For further details see S1 table. See acknowledgments for museum abbreviations.

Taxon	Sample Size (Males, Females, Unknown)	Museum Collection
**African colobines**		
*Colobus guereza*	(3,4,0)	KNM
*Colobus polykomos*	(0,1,0)	KNM
*Piliocolobus rufomitratus*	(2,1,1)	AMNH, KNM
*Piliocolobus kirkii*	(0,1,0)	KNM
**Asian colobines**		
*Nasalis larvatus*	(5,1,0)	AMNH, USNM
*Presbytis comata*	(0,2,0)	AMNH
*Presbytis melalophos*	(1,0,0)	AMNH
*Rhinopithecus roxellanae*	(1,0,0)	AMNH
*Semnopithecus johnii*	(1,0,0)	USNM
*Trachypithecus cristatus*	(1,1,0)	AMNH
*Tracypithecus obscurus*	(1,3,0)	AMNH
**Fossil Colobines**		
*Cercopithecoides williamsi*	(1,0,0)	KNM
*Mesopithecus pentelicus*	(0,2,2)	MNHN
**Cercopithecins**		
*Cercopithecus albogularis*	(1,3,0)	KNM
*Cercopithecus mitis*	(1,2,0)	AMNH, USNM
*Cercopithecus neglectus*	(1,0,0)	AMNH
*Chlorocebus aethiops*	(1,1,0)	AMNH, KNM
*Erythrocebus patas*	(2,0,0)	AMNH, KNM
**Papionins**		
*Cercocebus agilis*	(3,0,0)	AMNH
*Lophocebus albigena*	(1,1,0)	AMNH
*Macaca fascicularis*	(1,0,0)	USNM
*Macaca nemestrina*	(3,1,0)	USNM
*Macaca thibetana*	(6,0,0)	AMNH, USNM
*Mandrillus sphinx*	(0,1,0)	AMNH
*Papio hamadryas*	(4,1,1)	KNM, USNM
*Theropithecus gelada*	(3,3,0)	AMNH, KNM, HERC, NME

First, regression analyses were undertaken to establish the relationship between base area (medio-lateral width x dorso-palmar height) and proximo-distal length for each metacarpal. Simple indices of shape comparing the base area of MC I versus the base areas of the other MCs were then compared between extant African colobines (Colobina), extant Asian colobines (Presbytina), extant cercopithecines (Cercopthecinae), *Cercopithecoides williamsi*, and *Mesopithecus*, both graphically and statistically through one-way ANOVAs with Games-Howell post-hoc comparisons for significance due to the inequality of variances between groups. The ratios of MC I base area to the base areas of each of the other metacarpals were also tested for allometric scaling with hand size by conducting correlations against the geometric mean of 13 metacarpal measurements for each specimen (the 13 measurements preserved in KNM-ER 4420, explained further below). While MC I base area/MC II base area and MC I base area/MC V base area were not allometric (log MC I base area/MC II base area p = 0.08; PGLS p = 0.92; log MC I base area/MC V base area p = 0.06; PGLS p = 0.23), the other two ratios were determined to be slightly positively allometric (log MC I base area/MC III base area, p < 0.05; PGLS p = 0.48; log MC I base area/MC IV base area, p < 0.05, PGLS p = 0.43). Therefore, these last two ratios were also plotted relative to regression lines with 95% confidence intervals for each taxonomic group (cercopithecines, Asian colobines, and African colobines) to more accurately assess the distribution among taxa.

To specifically assess the size of MC I base area relative to the overall size of the hand rather than relative to other MC base areas, one-way ANOVAs were calculated between African colobines, Asian colobines and cercopithecines using a ratio of MC I base area divided by the geometric mean. Because this last ratio was also significantly correlated with hand size (i.e., positively allometric, p < 0.01; PGLS p < 0.01), regression equations were again calculated for African colobines, Asian colobines, and cercopithecines separately with 95% confidence intervals for individual data points plotted around each regression line.

Finally, an overall assessment of MC I—MC V morphology was provided by a principal components analysis (PCA) of 13 size-corrected variables for 72 specimens; raw measurements for each specimen were divided by the geometric mean of all 13 measurements for each specimen in order to control for absolute size variation among specimens. The 13 variables included in the PCA were those present in KNM-ER 4420: all 10 base diameters plus the proximo-distal lengths of MC III—V (S1 Table). Post-hoc correlation analyses were then performed to better determine which measures and features were most prominent in the loadings on the first 3 principal component (PC) axes.

## Results

**Fig 3:**
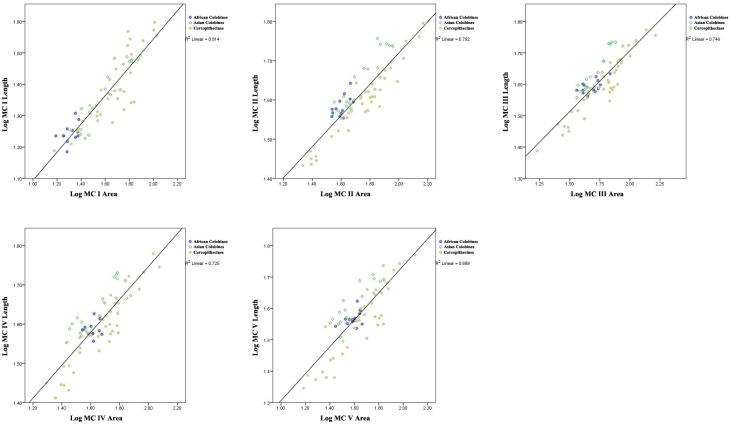
Bivariate plots showing correlation between metacarpal basal area and length transformed as logarithms.

Regression analyses demonstrate that the base area of a given metacarpal is highly positively correlated with its overall length in cercopithecoid primates (Fig 3; Table 3). All regressions of MC base area vs. length within the same element are highly significant, and most importantly for our analyses, the tightest correlation is between MC I base area and MC I length (*r* ^*2*^ = 0.814, PGLS *r* ^*2*^ = 0.660). In fact, the correlation coefficient between MC I base area and MC I length within colobines is even higher (*r* ^*2*^ = 0.872, PGLS *r* ^*2*^ = 0.801), demonstrating that most of the variation lies within cercopithecines and that MC I base area is an accurate predictor of MC I length within colobines.

**Table 3:** Results of correlation analyses for metacarpal lengths with base areas. All models were highly significant with p < 0.001.

Correlation Analysis	*n*	Regression equation	*r* ^*2*^-value	PGLS *r* ^*2*^-value
**MC I**	71	y = 0.45x + 0.64	0.814	0.660
**MC II**	71	y = 0.40x + 0.92	0.792	0.737
**MC III**	71	y = 0.39x + 0.93	0.746	0.749
**MC IV**	71	y = 0.42x + 0.91	0.725	0.689
**MC V**	69	y = 0.43x + 0.88	0.689	0.631

**Fig 4:**
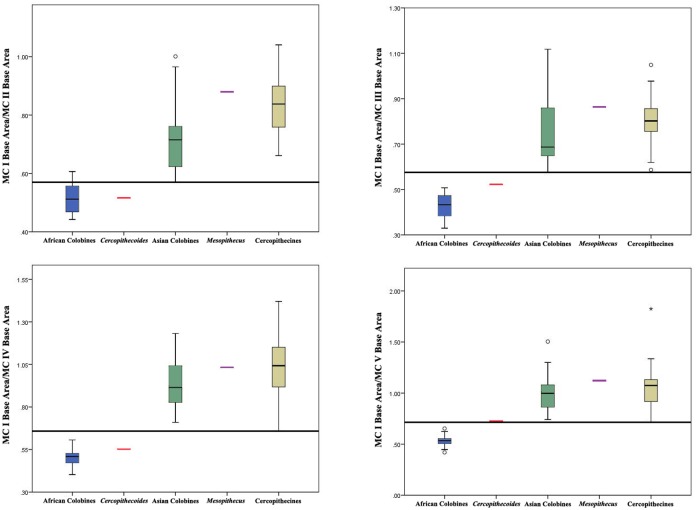
Box plots showing basal area of MC I compared to those of MC II-V. African colobines shown in blue, *Cercopithecoides* in red, Asian colobines in green, *Mesopithecus* in purple, and cercopithecines shown in gold. Cercopithecini and Papionini pooled within the Cercopithecinae as they are not significantly different in these measures. The central bar represents the median, or 50th percentile. The bottom and top of each box represent the value at the 25th and 75th percentiles, respectively, and the whiskers extend to the farthest observation that is less than 1.5 times the length of the box. Any individuals outside of the whisker range are marked separately.

All of the ANOVAs are significant for the ratios of MC I base area/MC II base area, MC I base area/MC III base area, MC I base area/MC IV base area, MC I base area/MC V Area, and MC I base area/Geometric Mean, with post-hoc tests suggesting that African colobines < Asian colobines < cercopithecines for MC I base area/MC II base area and MC I base area/Geometric Mean (Table 4). ANOVAs for MC I base area/MC III base area, MC I base area/MC IV base area, and MC I base area/MC V base area do not show a significant difference between Asian colobines and cercopithecines, although African colobines remain statistically different from both. This suggests that MC I base area/MC II base area and MC I base area/Geometric Mean are probably the most informative measures, since Asian colobines are indeed noted to have at least somewhat reduced thumbs compared to cercopithecines [4, 28]. This is perhaps not surprising since MC I base area and MC II base area are the two measures most highly correlated with their respective MC lengths. Still, boxplots of all metacarpal ratios illustrate that *C*. *williamsi* consistently falls within the range of African colobines and outside the ranges of Asian colobines and cercopithecines while *Mesopithecus* falls within the ranges of both Asian colobines and cercopithecines (Fig 4).

**Table 4:** Results of ANOVAs for MC I basal area compared to those of MC II—V.

Feature/Ratio	Taxon	*n*	Mean, s.d.	Range	Significance	PGLS Significance
MC I Base Area / MC II Base Area	**African colobines**	13	0.51, 0.05	0.44–0.61	*<* Asian colobines, *p* < 0.001 < Cercopithecines, *p* < 0.001	*<* Asian colobines, *p* < 0.001 < Cercopithecines, *p* < 0.001
	**Asian colobines**	17	0.72, 0.14	0.57–1.00	*>* African colobines, *p* < 0.001 < Cercopithecines, *p* < 0.05	*> African colobines*, *p* < 0.001 *< Cercopithecines*, *p* < 0.001
	**Cercopithecines**	41	0.83, 0.10	0.66–1.04	*>* African colobines, *p* < 0.001 > Asian colobines, *p* < 0.05	*> African colobines*, *p* < 0.001 *> Asian colobines*, *p < 0*.*001*
MC I Base Area / MC III Base Area	**African colobines**	13	0.43, 0.05	0.33–0.51	*<* Asian colobines, *p* < 0.001 < Cercopithecines, *p* < 0.001	*< Asian colobines*, *p* < 0.001 *< Cercopithecines*, *p* < 0.001
	**Asian colobines**	17	0.75, 0.15	0.58–1.12	*>* African colobines, *p* < 0.001 Cercopithecines, *p* = 0.31	*> African colobines*, *p* < 0.001 *< Cercopithecines*, *p* = 0.56
	**Cercopithecines**	41	0.81, 0.10	0.59–1.05	*>* African colobines, *p* < 0.00 > Asian colobines, *p* = 0.31	*> African colobines*, *p* < 0.001 *> Asian colobines*, *p* = 0.56
MC I Base Area / MC IV Base Area	**African colobines**	13	0.50, 0.06	0.40–0.61	*<* Asian colobines, *p* < 0.001 < Cercopithecines, *p* < 0.001	*< Asian colobines*, *p < 0*.*001 < Cercopithecines*, *p < 0*.*001*
	**Asian colobines**	17	0.93, 0.15	0.71–1.23	*>* African colobines, *p* < 0.001 < Cercopithecines, *p* = 0.06	*> African colobines*, *p < 0*.*001 < Cercopithecines*, *p* = 0.57
	**Cercopithecines**	41	1.03, 0.17	0.66–1.42	*>* African colobines, *p* < 0.001 > Asian colobines, *p* = 0.06	*> African colobines*, *p < 0*.*001 > Asian colobines*, *p* = 0.57
MC I Base Area / MC V Base Area	**African colobines**	13	0.53, 0.07	0.42–0.65	*<* Asian colobines, *p* < 0.001 < Cercopithecines, *p* < 0.001	*< Asian colobines*, *p < 0*.*001 < Cercopithecines*, *p < 0*.*001*
	**Asian colobines**	17	1.02, 0.21	0.74–1.50	*>* African colobines, *p* < 0.001 < Cercopithecines, *p* = 0.76	*> African colobines*, *p < 0*.*001 < Cercopithecines*, *p* = 0.51
	**Cercopithecines**	39	1.06, 0.19	0.71–1.82	*>* African colobines, *p* < 0.00 > Asian colobines, *p* = 0.76	*> African colobines*, *p < 0*.*001 > Asian colobines*, *p* = 0.51
MC I Base Area / Geometric Mean	**African colobines**	13	2.23, 0.21	1.83–2.53	*<* Asian colobines, *p* < 0.001 < Cercopithecines, *p* < 0.001	*< Asian colobines*, *p* < 0.01 *< Cercopithecines*, *p* < 0.05
	**Asian colobines**	17	3.88, 1.02	2.76–5.90	*>* African colobines, *p* < 0.001 < Cercopithecines, *p* < 0.05	*> African colobines*, *p* < 0.01 *< Cercopithecines*, *p* = 0.70
	**Cercopithecines**	41	4.62, 1.08	2.36–7.21	*>* African colobines, *p* < 0.001 > Asian colobines, *p* < 0.05	*> African colobines*, *p* < 0.05 *> Asian colobines*, *p* = 0.70

**Fig 5:**
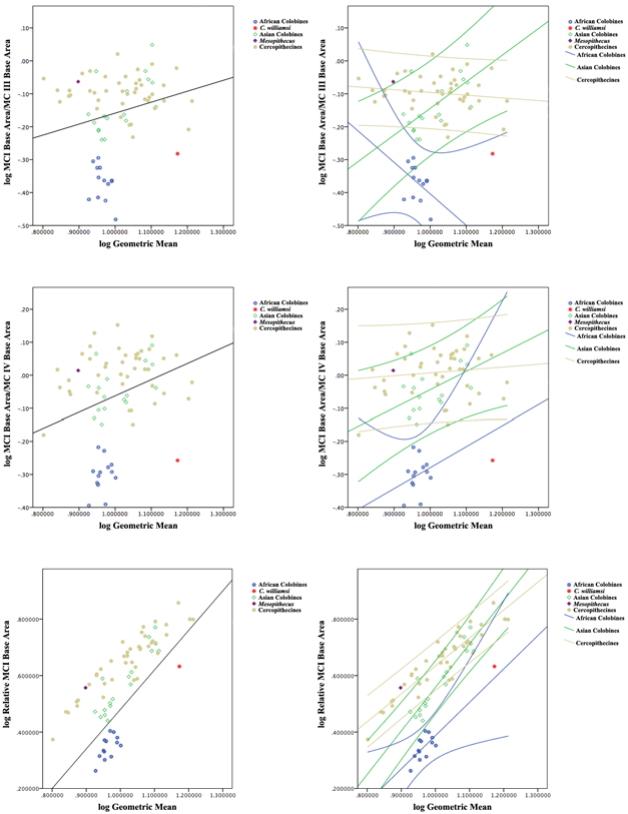
Regression plots of allometrically influenced MC I base area ratios compared to the geometric mean. In each panel, the overall cercopithecoid regression is presented on the left and the subgroup regression lines with 95% confidence intervals are presented on the right. Note that in all comparisons, *C*. *williamsi* falls within the 95% confidence interval of African colobines, but outside the 95% confidence intervals of Asian colobines and cercopithecines.

Games-Howell post-hoc comparisons used due to unequal variances. n = sample size, PGLS = phylogenetic least squares significance values. Ratios for *Cercopithecoides williamsi* are: MC I/MC II = 0.52, MC I/MC III = 0.52, MC I/MC IV = 0.55, MC I/MC V = 0.73, MC I/Geometric Mean = 4.29. Note that MC V of *C*. *williamsi* is slightly damaged and abraded, resulting in a likely underestimate of MC V base area and an overestimate of MC I/MC V for this specimen. Ratios for *Mesopithecus pentelicus* are: MC I/MC II = 0.88, MC I/MC III = 0.86, MC I/MC IV = 1.03, MC I/MC V = 1.12, MC I/Geometric Mean = 3.60. For boxplots with ranges, see also Fig 4. For Relative MC I base area regressions with 95% confidence intervals, see Fig 5.

To more closely examine those ratios scaling allometrically (see Table 5 for equations and *r* ^*2*^ values), regression plots are presented in Fig 5. Regression plots of MC I base area/ MC III base area and MC I base area/MC IV base area demonstrate that *C*. *williamsi* falls outside of the 95% confidence interval of Asian colobines and cercopithecines, but within the 95% confidence interval for African colobines. A regression plot of relative MC I base area (MC I base area/Geometric Mean) vs. Geometric Mean demonstrates that only African colobines and *C*. *williamsi* fall below the regression line, and again *C*. *williamsi* falls within the 95% confidence interval of African colobines but outside the 95% confidence limits of both Asian colobines and cercopithecines. Thus, for all ratios, *C*. *williamsi* exhibits a relative MC I base area within the range that would be predicted for an African colobine of its size, but outside of the range predicted for an Asian colobine or cercopithecine of its size. *Mesopithecus* falls outside the range of African colobines in all cases, outside the 95% confidence interval of Asian colobines in most cases, and within the 95% confidence interval of cercopithecines in all cases.

**Table 5:** Results of correlation analyses for allometrically influenced base area ratios.

Correlation Analysis	*n*	Regression equation	*r* ^*2*^-value	PGLS *r* ^*2*^-value
**MC I Base Area/MC III Base Area vs. Geometric Mean**	71	y = 0.33x - 0.49	0.055	0.007
**MC I Base Area/MC IV Base Area vs. Geometric Mean**	71	y = 0.49x - 0.55	0.093	0.009
**Relative MC I Base Area vs. Geometric Mean**	71	y = 1.34x - 0.77	0.557	0.592

See Materials and Methods section of text for *p*-values of each regression and Fig 5 for graphical representation.

The PCA of 13 size-adjusted metacarpal variables reveals consistent differences in the relative proportions of the metacarpals among cercopithecid taxa. The first three PCs explain 93% of the total variance in the sample (Table 6). Higher order PCs explain little variance and neither correlate significantly with size nor strongly segregate taxonomic groups and therefore are not considered further here.

**Table 6:** Eigenvalues, percent variance explained, and summary of loadings for first 4 principal components.

Principal Component	Eigenvalue	% Variance	Loadings: strongly positive vs. negative
**PC 1**	0.283	85%	MC3-5 lengths vs. Basal Diameters
**PC 2**	0.0170	5.1%	MC5 vs. MC3-4 lengths
**PC 3**	0.0113	3.4%	MC3-5 vs MC1 basal diameters
**PC 4**	0.00603	1.8%	MC1-5 basal DP vs. ML

**Fig 6:**
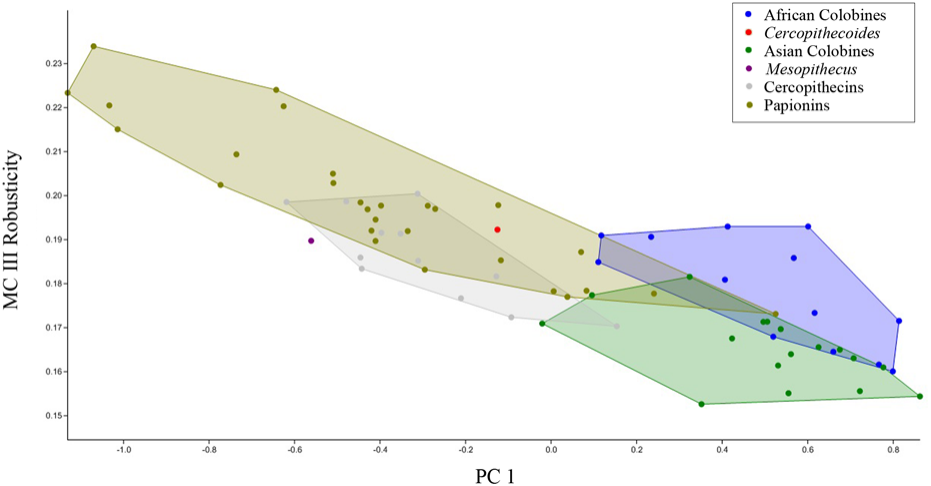
Scatter plot of MC III robusticity vs. scores of PC1. Metacarpal robusticity is calculated as the Basal Area (Dorsopalmar x Mediolateral Diameters) / Length. PC1 based on PCA of 13 size adjusted variables as described in text.

The first principal component (PC1) accounts for a full 85% of the sample variance and loads strongly positively on MC III—V lengths (the only length measures in the analysis) and moderately negatively on MC I—V base diameters (Table 6, and S2 Table). It accounts for such a large portion of the total variance because it represents much of the variation in metacarpal proximodistal length, and the length measurements are absolutely much larger than the proximal diameters and therefore vary much more. PC1 separates most colobines at the positive end from cercopithecines which are at the negative end. Size, as represented by the geometric mean of all 13 variables, is correlated with PC1 (p = .04), but it explains little of the variance (r^2^ = 0.06). Metacarpal robusticity (length relative to base diameter) shows a much stronger correlation with PC1 (p < 0.0001, r^2^ = 0.75) (Fig 6). Metacarpal robusticity has been shown to reflect locomotor mode and substrate preference more than taxonomic groupings [31]. The PC1 score for *Cercopithecoides williamsi* falls in the cercopithecine range, but is near colobines, reflecting its more robust metacarpals compared with other colobines. The PC1 score for *Mesopithecus pentelicus* falls well within the cercopithecine range and much farther from the other colobines (Fig 6).

**Fig 7:**
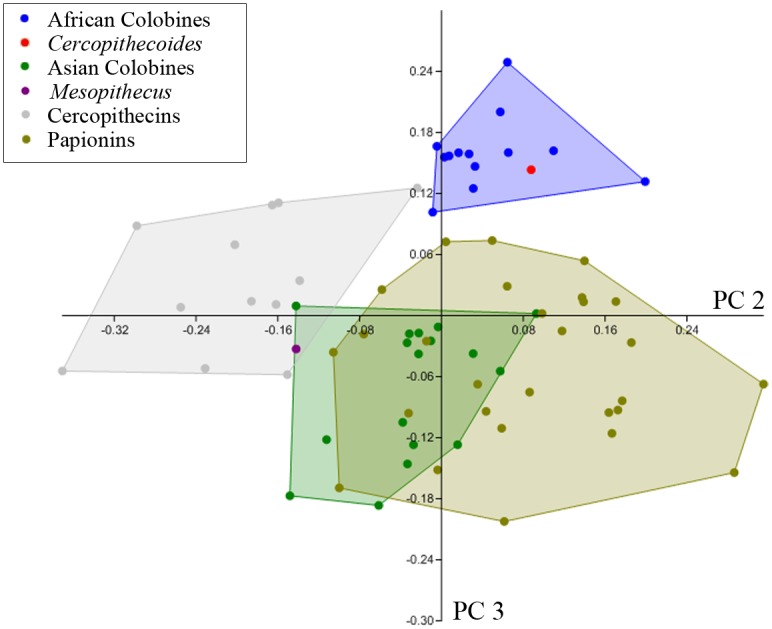
Scatter plot of scores of PC2 vs. PC3 based on PCA of 13 size adjusted variables as described in text.

The second principal component accounts for 5% of variance and loads strongly positively on MC V length and negatively on MC III—IV lengths. The second PC is also correlated with size (p = 0.0001, r^2^ = 0.38). PC2 separates the Cercopithecini at the negative end (i.e., they have short fifth metacarpals) from all others, but particularly the Papionini at the positive end as their metacarpals III—V are more even in length (Fig 7). To a lesser degree, PC2 also separates Colobina at the positive end from Presbytina which are more negative (i.e., they have shorter fifth metacarpals). The PC2 score for *Cercopithecoides williamsi* is within the colobinan range, while that for *Mesopithecus pentelicus* falls in the narrow overlap zone of the Presbytina and Cercopithecini (Fig 7).

The third principal component accounts for 3% of the total variance and loads positively on the base diameters of MC III—V and negatively on those of MC I. It is also correlated with size (p = 0.027; r^2^ = 0.07). It places the colobinans at the positive end, clearly separated from all presbytinans and papionins, but overlapping the cercopithecins slightly (Fig 7). The PC3 score for *Cercopithecoides williamsi* is within the African colobine range, whereas that for *Mesopithecus* lies at a level where the ranges of the presbytinans, cercopithecins and papionins overlap (Fig 7).

## Discussion

**Fig 8:**
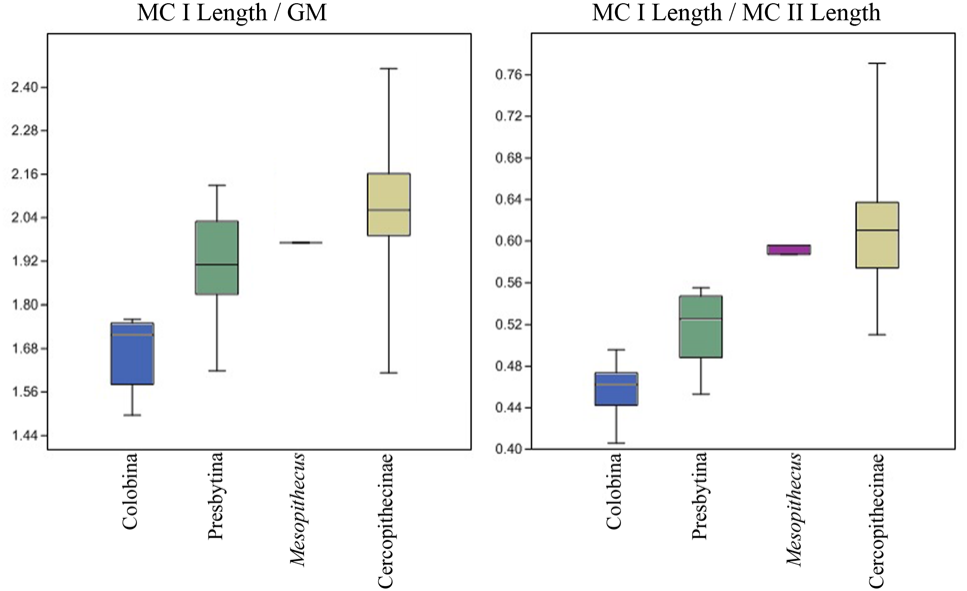
Box plots showing relative length of MC I. On the left MC I PD Length / Geometric Mean of 13 variables. On the right MC I PD Length / MC II PD Length. Colors and groupings as in Fig 4.

Our data clearly corroborate previous analyses showing that African colobines have significant reduction in the size of the MC I compared to cercopithecines and presbytinans, with the length of the MC I in the latter group intermediate between those of colobinans and cercopithecines [23,28](Fig 8). Interestingly, in terms of the relative size of MC I base area, the same relationship between the three groups is recovered in comparisons of MC I base area compared to the geometric mean and MC I base area compared to MC II base area, but there is little difference between Asian colobines and cercopithecines when MC I base area is compared to the MC III-V base areas (Fig 4). *Mesopithecus* is within the upper size range for both presbytinans and cercopithecines in MC I base area compared to MC II, IV, and V, but within only the cercopithecine range for MC I length, MC I base area compared to MC III base area, and MC I base area compared to the geometric mean of all metacarpal measurements [23,28] (Figs 4, 5 and 8). Thus, there is no evidence for pollical reduction in *Mesopithecus* based on metacarpal base proportions. Furthermore, the degree of MC I reduction in base area found in all extant African colobines is greater than in all cercopithecines and Asian colobines studied, suggesting there is no overlap in this feature and marking it as a good character for discriminating the extant tribes, particularly when compared to the MC II base area or overall hand size as represented by the geometric mean. Among extant primates, *Ateles* and *Brachyteles* also have reduced thumbs [6,28,32]. In these platyrrhines, it is associated with the frequent use of suspensory locomotor and positional behaviors in an arboreal setting, which may suggest that the inferred Late Miocene last common ancestor of the colobinans would have been arboreal (and possibly engaged in some suspensory behaviors), as suggested by Nakatsukasa et al. [23] from their analysis of *Microcolobus*.

Almost 40 years ago, Delson [24; p. 205] proposed that when thumbs of *Cercopithecoides*, *Paracolobus* and *Libypithecus* were recovered, they would be reduced similar to those of extant African colobines. In fact, KNM-ER 4420 clearly has a degree of MC I reduction comparable to extant African colobines, thus implying that *Cercopithecoides williamsi* is a true colobinan and marking the earliest appearance of colobinan thumb reduction in the fossil record. More specifically, the reduced thumb of *C*. *williamsi* places the timing of colobinan-level pollical reduction between the divergence of the African and Asian clades (11–8 Ma) and the divergence of the crown colobinans (9–6 Ma) [7,9,10]. This phylogenetic inference is also congruent with previous observations that the P^3^ protocone is greatly reduced or absent in *Cercopithecoides*, another feature shared by all extant Afrian colobines [4,17,19,21,33–35]. Based on these features alone, it is unclear whether *Cercopithecoides* is a stem colobinan or a member of the crown group more specifically related to one of the extant genera, *Procolobus* or *Colobus*. Given that molecular estimates suggest *Colobus* and *Procolobus* diverged 9–6 Ma, the Pliocene through Pleistocene age of *Cercopithecoides* suggests that the latter interpretation is certainly possible [9].

There are some morphological features that favor *Cercopithecoides* being a stem colobinan. First, *Cercopithecoides* in general, and *C*. *williamsi* in particular, has a suite of postcranial features that suggest it was adapted for terrestrial locomotion, making it different from both *Colobus* and *Procolobus* [18,19,21]. If, however, the ancestral colobinan was arboreal as posited above, this would imply that *Cercopithecoides* is secondarily terrestrial, which could therefore be an autapomorphy not excluding the fossil taxon from a sister relationship to either *Colobus* or *Procolobus*.

A second feature supporting *Cercopithecoides* as a stem colobinan is the presence of a maxillary sinus, a feature absent among all extant colobines, but present in at least *C*. *williamsi* and *C*. *kimeui* [36]. A maxillary sinus is also present in the North African species *Libypithecus markgrafi* [36], possibly suggesting a close phylogenetic relationship between these two genera. On the other hand, a maxillary sinus is known in all extant species of the papionin *Macaca* and some related fossil forms, implying at least two independent reacquisitions of this primitive mammalian feature apparently lost in the common ancestor of Cercopithecoidea [36]; it is thus possible that the occurrences in *Libypithecus* and *Cercopithecoides* were convergent as well.

If, however, *Cercopithecoides* is more closely related to one of the extant genera, a link to *Procolobus* seems more likely, though the evidence is not overwhelming. In particular, the mandible of *Cercopithecoides* resembles that of *Procolobus*, especially *P*. *verus*. Both genera have relatively shallow, but robust and thick, corpora with well-developed *prominentia laterales* [14,37,38]. Furthermore, both genera lack the expanded gonial region of *Colobus*. Finally, several *Cercopithecoides* species (*C*. *williamsi*, *C*. *kimeui*, *C*. *haasgati*) have mandibular symphyses marked by a median mental foramen [14,19,34], a feature otherwise found only in *P*. *verus* among extant colobines, where it is variably present [39]. If this phylogenetic position of *Cercopithecoides* is correct, it again requires the terrestriality of *Cercopithecoides* to be autapomorphic. The maxillary sinus would therefore be either autapomorphic or shared with *Libypithecus*, but both genera would be more closely related to *Procolobus* than to *Colobus*. This latter hypothesis may find some support in the presence of a distinct sagittal crest in *Libypithecus*, as in *Procolobus* but unlike *Cercopithecoides*.

Ultimately, deciphering the best phylogenetic placement of *Cercopithecoides* within the colobinans must await a more complete analysis that considers a wider array of characters and includes more of the extant and fossil genera. Nonetheless, the manual morphology of KNM-ER 4420, in combination with that previously known for *Mesopithecus* and *Microcolobus*, provides significant insight into the evolutionary history of colobines. The unreduced MC I of *Microcolobus*, along with the relatively low cusps and other possibly primitive features support the interpretation that this genus is a stem colobine [23,26]. This phylogenetic placement, along with the African occurrence of both the earliest colobine fossils [23,26,40] and victoriapithecids [41] all place the origin of colobines in Africa. Furthermore, this also suggests that presbytinans likely migrated to Asia from Africa. The relatively large pollex of *Mesopithecus* [23,27,28,42] would be most compatible with its position as either a stem colobine or a stem presbytinan. However, if *Mesopithecus* is a stem colobine, it implies that presbytinans represent a second colobine migration out of Africa.

Given the evidence at hand, it is difficult to determine if thumb reduction in the Asian and African colobine lineages evolved in parallel or is homologous as previously suggested [4,17]. If *Mesopithecus* is a stem colobine, then it is probable that thumb reduction is a shared derived character uniting all crown colobines and that Asian colobines retain a more modest degree of pollical reduction present in the common ancestor of all living colobines. In this scenario, African colobines have simply become even more derived relative to the common ancestor in terms of metacarpal and pollical reduction. If, however, *Mesopithecus* is a stem presbytinan, as is often assumed on biogeographical and general morphological grounds [2,4,5,17], then only one migration out of Africa is necessary, but independent evolution of pollical reduction in the two extant colobine subtribes is most likely [23]. In our analyses, *Mesopithecus* shows no evidence of first metacarpal reduction and is most often more similar to cercopithecines than to Asian colobines, especially in MC I length and relative MC I base area.

## Conclusions

Our analyses of *Cercopithecoides williamsi* (KNM-ER 4420) metacarpal morphology demonstrate that this fossil colobine had evolved a significantly reduced pollex, the first good evidence for colobine thumb reduction known so far in the fossil record. Among cercopithecoid monkeys, extremely reduced thumbs are only found in living African colobines, making it almost certain that *C*. *williamsi* is a close phylogenetic relative of extant *Colobus* and *Procolobus*. Because a reduced thumb is generally thought to be associated with highly arboreal behavior such as suspensory locomotion, the thumb reduction observed in *C*. *williamsi* suggests that the terrestrial features in other parts of its skeleton are secondarily derived from a more arboreal ancestor. Finally, early putative fossil Asian colobines such as *Mesopithecus* show no evidence of reduced pollices, making it possible that thumb reduction in living Asian and African colobines evolved convergently.

## Supporting Information

S1 Table Data used in this analysis. * = Measurement not included in the calculation of the geometric mean and also excluded from the principal components analysis.
(DOC)

S2 Table Loadings from principal components analysis. (DOCX)

S1 PGLSTree Tree file used for phylogenetic least squares adjustments in regressions and ANOVAs. Tree file in nexus format.
(NEX)
