# Supplementary material for: The Hand of Cercopithecoides williamsi (Mammalia, Primates): Earliest Evidence for Thumb Reduction among Colobine Monkeys
Source: PLoS One. 2015 May 20;10(5):e0125030. doi: 10.1371/journal.pone.0125030 (PMC4439063; doi:10.1371/journal.pone.0125030)
Supplement: S1 Table — * = Measurement not included in the calculation of the geometric mean and also excluded from the principal components analysis. (DOC) [file pone.0125030.s001.doc]

**Table S1.** Data used in this analysis. * = Measurement not included in the calculation of the geometric mean and also excluded from the principal components analysis.

|  |  |  |  |  |  | **Metacarpal 1** | | | **Metacarpal 2** | | | **Metacarpal 3** | | | **Metacarpal 4** | | | **Metacarpal 5** | | |
| --- | --- | --- | --- | --- | --- | --- | --- | --- | --- | --- | --- | --- | --- | --- | --- | --- | --- | --- | --- | --- |
| **Institution** | **Catalog No.** | **Species** | **Taxon** | **Sex** | **Observer** | **Length*** | **Base PD** | **Base ML** | **Length*** | **Base PD** | **Base ML** | **Length** | **Base PD** | **Bael ML** | **Length** | **Base PD** | **Base ML** | **Length** | **Base PD** | **Base ML** |
|  |  |  |  |  |  |  |  |  |  |  |  |  |  |  |  |  |  |  |  |  |
| KNM | OM 3069 | Colobus guereza kikuyuensis | Colobina | Female | CG | 17.9 | 4.6 | 4.6 | 36.1 | 7.5 | 5.2 | 36.5 | 7.7 | 5.8 | 36.0 | 6.6 | 6.3 | 34.4 | 6.1 | 6.8 |
| KNM | OM 5088 | Colobus guereza kikuyuensis | Colobina | Female | CG | 17.2 | 4.5 | 4.0 | 37.4 | 7.4 | 5.5 | 39.9 | 7.4 | 5.5 | 39.1 | 6.6 | 5.5 | 37.1 | 6.2 | 6.5 |
| KNM | OM 3065 | Colobus guereza kikuyuensis | Colobina | Female | CG | 16.5 | 4.2 | 4.6 | 35.8 | 7.5 | 5.5 | 37.7 | 7.9 | 6.5 | 37.5 | 7.2 | 6.6 | 35.5 | 6.4 | 7.2 |
| KNM | OM 3091 | Colobus guereza kikuyuensis | Colobina | Female | CG | 18.1 | 4.7 | 4.1 | 36.8 | 7.5 | 5.3 | 38.1 | 6.9 | 5.9 | 37.6 | 6.8 | 6.0 | 35.6 | 5.0 | 7.0 |
| KNM | OM 3118 | Colobus guereza kikuyuensis | Colobina | Male | CG | 17.9 | 4.4 | 4.8 | 37.8 | 7.2 | 5.1 | 39.5 | 7.3 | 5.7 | 38.5 | 6.7 | 5.2 | 36.4 | 5.8 | 6.9 |
| KNM | OM 3088 | Colobus guereza kikuyuensis | Colobina | Male | CG | 17.0 | 4.9 | 4.6 | 39.4 | 7.7 | 6.3 | 42.1 | 8.3 | 6.3 | 42.3 | 7.5 | 5.6 | 39.2 | 6.1 | 7.3 |
| KNM | OM 3119 | Colobus guereza matschiei | Colobina | Male | CG | 17.7 | 4.7 | 5.0 | 40.0 | 8.2 | 5.6 | 40.9 | 8.1 | 6.7 | 41.1 | 7.2 | 6.4 | 38.3 | 6.2 | 7.1 |
| KNM | OM 6364 | Colobus polykomos | Colobina | Female | CG | 17.2 | 3.9 | 4.6 | 36.9 | 7.1 | 4.9 | 38.1 | 7.1 | 5.1 | 38.5 | 7.0 | 5.0 | 36.1 | 6.4 | 6.0 |
| AMNH | 52334 | Piliocolobus rufomitratus | Colobina | Female | CG | 17.2 | 5.5 | 4.3 | 39.5 | 6.5 | 6.0 | 38.6 | 8.4 | 6.5 | 39.3 | 6.9 | 5.8 | 36.7 | 5.1 | 7.1 |
| KNM | OM 3019 | Piliocolobus kirkii | Colobina | Female | CG | 17.2 | 4.3 | 3.6 | 36.1 | 7.0 | 4.9 | 37.3 | 6.8 | 6.0 | 37.6 | 6.4 | 6.0 | 34.9 | 4.9 | 5.7 |
| AMNH | 52298 | Piliocolobus rufomitratus | Colobina | Male | CG | 19.4 | 4.9 | 4.9 | 41.3 | 7.5 | 5.6 | 39.6 | 8.0 | 7.1 | 38.3 | 6.9 | 6.6 | 36.7 | 5.5 | 7.0 |
| KNM | OM 4356 | Piliocolobus rufomitratus | Colobina | Male | CG | 15.3 | 4.8 | 4.0 | 37.7 | 7.5 | 4.6 | 38.2 | 7.8 | 6.4 | 37.7 | 7.1 | 5.8 | 36.8 | 5.8 | 5.8 |
| AMNH | 52303 | Piliocolobus rufomitratus | Colobina | Unknown | CG | 20.3 | 4.8 | 4.7 | 43.9 | 7.7 | 6.0 | 43.0 | 9.0 | 7.6 | 41.6 | 7.2 | 6.4 | 42.0 | 6.1 | 6.9 |
| AMNH | 103669 | Nasalis larvatus | Presbytina | Female | CG | 26.5 | 5.8 | 7.2 | 47.9 | 8.7 | 6.6 | 47.1 | 8.0 | 7.6 | 45.1 | 7.5 | 6.6 | 42.2 | 5.3 | 6.1 |
| AMNH | 106273 | Nasalis larvatus | Presbytina | Male | CG | 30.7 | 7.0 | 8.6 | 55.3 | 10.4 | 7.9 | 53.5 | 8.9 | 7.7 | 52.5 | 8.5 | 6.8 | 49.5 | 6.8 | 8.5 |
| AMNH | 106275 | Nasalis larvatus | Presbytina | Male | CG | 29.7 | 7.7 | 8.2 | 54.7 | 11.0 | 8.2 | 52.7 | 9.9 | 8.8 | 51.4 | 10.0 | 6.9 | 48.6 | 7.3 | 8.9 |
| AMNH | 103670 | Nasalis larvatus | Presbytina | Male | CG | 30.0 | 7.7 | 8.6 | 57.1 | 10.3 | 6.9 | 54.3 | 9.0 | 7.9 | 53.2 | 8.8 | 6.8 | 48.8 | 6.2 | 7.1 |
| AMNH | 103671 | Nasalis larvatus | Presbytina | Male | CG | 29.9 | 7.8 | 8.4 | 54.8 | 10.9 | 7.9 | 54.2 | 9.3 | 8.2 | 53.8 | 9.5 | 6.4 | 51.1 | 6.9 | 8.2 |
| USNM | 198276 | Nasalis larvatus | Presbytina | Male | EG | 30.0 | 8.1 | 9.2 | 55.2 | 9.9 | 7.6 | 53.7 | 8.7 | 7.7 | 52.0 | 8.5 | 7.2 | 49.3 | 7.7 | 9.0 |
| AMNH | 102462 | Presbytis comata | Presbytina | Female | KP | 21.1 | 5.3 | 5.9 | 40.6 | 7.8 | 7.0 | 42.0 | 7.3 | 6.4 | 41.3 | 6.3 | 5.1 | 38.7 | 5.1 | 5.9 |
| AMNH | 102047 | Presbytis comata | Presbytina | Female | KP | 21.0 | 4.6 | 5.5 | 38.4 | 7.4 | 5.9 | 38.8 | 7.2 | 6.1 | 38.5 | 6.6 | 5.4 | 37.2 | 5.6 | 6.1 |
| AMNH | 106599 | Presbytis melalophos | Presbytina | Male | KP | 20.5 | 5.3 | 5.6 | 40.6 | 8.3 | 5.0 | 41.3 | 7.4 | 5.9 | 40.3 | 6.4 | 5.4 | 39.3 | 5.4 | 6.2 |
| AMNH | 119648 | Rhinopithecus roxellanae | Presbytina | Male | KP | 23.3 | 6.4 | 6.9 | 42.4 | 8.9 | 7.7 | 43.3 | 8.0 | 6.8 | 41.8 | 7.3 | 7.0 | 39.6 | 6.4 | 6.9 |
| USNM | 49701 | Semnopithecus johnii | Presbytina | Male | EG | 24.3 | 6.4 | 6.2 | 45.6 | 7.8 | 6.3 | 47.2 | 8.4 | 7.2 | 46.2 | 7.5 | 6.5 | 43.5 | 6.3 | 7.3 |
| AMNH | 102461 | Trachypithecus cristatus | Presbytina | Female | KP | 17.4 | 4.6 | 5.4 | 37.3 | 6.9 | 5.5 | 37.8 | 6.6 | 5.8 | 36.8 | 6.2 | 5.4 | 35.5 | 5.7 | 5.2 |
| AMNH | 101504 | Trachypithecus cristatus | Presbytina | Male | KP | 16.9 | 5.1 | 5.3 | 37.3 | 7.2 | 6.5 | 39.1 | 7.6 | 5.8 | 37.2 | 6.5 | 5.3 | 35.8 | 4.6 | 5.5 |
| AMNH | 119492 | Trachypithecus obscurus | Presbytina | Female | KP | 19.1 | 5.0 | 5.2 | 39.1 | 6.7 | 6.8 | 38.3 | 6.9 | 6.1 | 38.7 | 4.9 | 5.8 | 35.8 | 5.8 | 5.9 |
| AMNH | 112976 | Trachypithecus obscurus | Presbytina | Female | KP | 19.2 | 5.3 | 6.5 | 39.3 | 7.0 | 5.1 | 39.5 | 6.5 | 5.7 | 39.9 | 5.3 | 5.6 | 36.8 | 5.0 | 5.3 |
| AMNH | 119496 | Trachypithecus obscurus | Presbytina | Female | KP | 18.0 | 5.0 | 5.0 | 36.3 | 6.7 | 5.2 | 36.1 | 6.6 | 5.5 | 35.7 | 5.2 | 5.2 | 32.2 | 5.6 | 5.5 |
| AMNH | 112977 | Trachypithecus obscurus | Presbytina | Male | KP | 17.3 | 5.1 | 5.7 | 37.8 | 7.4 | 6.3 | 39.1 | 7.3 | 6.9 | 38.6 | 6.8 | 5.4 | 36.0 | 5.1 | 6.0 |
| KNM | OM 6131 | Cercopithecus albogularis | Cercopithecini | Female | CG | 16.2 | 4.5 | 4.6 | 27.3 | 5.9 | 4.2 | 27.4 | 5.2 | 5.3 | 25.8 | 4.8 | 4.7 | 23.6 | 4.1 | 4.7 |
| KNM | OM 2797 | Cercopithecus albogularis | Cercopithecini | Female | CG | 17.5 | 4.8 | 5.1 | 27.9 | 6.4 | 4.2 | 28.2 | 6.1 | 5.1 | 27.0 | 5.6 | 5.0 | 24.0 | 4.6 | 5.1 |
| KNM | OM 4232 | Cercopithecus albogularis | Cercopithecini | Female | CG | 18.1 | 4.7 | 5.1 | 28.6 | 6.5 | 4.1 | 29.0 | 5.9 | 5.2 | 27.9 | 5.4 | 4.6 | 25.0 | 4.3 | 5.1 |
| USNM | 452550 | Cercopithecus mitis stuhlmanni | Cercopithecini | Female | EG | 17.8 | 4.8 | 4.9 | 28.2 | 6.1 | 4.0 | 29.2 | 5.8 | 5.0 | 27.8 | 6.0 | 4.3 | 24.0 | 5.1 | 5.4 |
| USNM | 452557 | Cercopithecus mitis stuhlmanni | Cercopithecini | Female | EG | 17.9 | 4.2 | 4.9 | 29.5 | 6.3 | 3.9 | 29.7 | 5.8 | 4.4 | 28.2 | 5.2 | 3.8 | 24.4 | 4.5 | 3.7 |
| AMNH | 52368 | Cercopithecus mitis stuhlmanni | Cercopithecini | Male | CG | 20.5 | 5.2 | 6.6 | 33.2 | 7.2 | 5.3 | 32.8 | 6.7 | 5.5 | 31.2 | 5.5 | 5.1 | 28.5 | 5.4 | 5.9 |
| KNM | OM 2791 | Cercopithecus albogularis | Cercopithecini | Male | CG | 21.5 | 5.4 | 5.5 | 35.9 | 8.1 | 5.4 | 35.5 | 6.8 | 6.1 | 34.6 | 6.1 | 5.5 | 31.3 | 5.1 | 6.3 |
| AMNH | 52429 | Cercopithecus neglectus | Cercopithecini | Male | EG | 20.2 | 5.1 | 5.9 | 32.2 | 7.1 | 4.8 | 32.5 | 6.2 | 5.3 | 31.1 | 6.1 | 4.2 | 27.6 | 4.9 | 5.5 |
| KNM | OM 7522 | Chlorcebus aethiops | Cercopithecini | Female | SF | 15.4 | 3.9 | 3.9 | 27.1 | 6.0 | 3.6 | 24.4 | 5.1 | 3.3 | 25.8 | 5.3 | 4.3 | 22.2 | 4.1 | 3.7 |
| AMNH | 167679 | Chlorcebus aethiops | Cercopithecini | Male | CG | 21.5 | 5.4 | 6.2 | 35.7 | 7.4 | 5.4 | 35.9 | 7.0 | 6.3 | 33.7 | 6.2 | 5.4 | 29.9 | 5.8 | 6.0 |
| KNM | OM 5093 | Erythrocebus patas | Cercopithecini | Male | CG | 26.0 | 6.2 | 7.0 | 43.7 | 10.1 | 6.5 | 43.4 | 10.0 | 7.4 | 42.4 | 9.0 | 6.8 | 38.0 | 7.2 | 6.7 |
| AMNH | 34712 | Erythrocebus patas | Cercopithecini | Male | EG | 22.6 | 6.7 | 6.3 | 41.8 | 10.5 | 5.9 | 40.9 | 8.7 | 7.6 | 39.8 | 8.8 | 6.1 | 37.5 | 8.0 | 7.3 |
| AMNH | 52641 | Cercocebus agilis | Papionini | Male | EG | 23.9 | 6.4 | 7.5 | 39.0 | 8.3 | 6.8 | 38.9 | 7.3 | 7.6 | 37.9 | 6.8 | 5.8 | 36.4 | 6.2 | 7.0 |
| AMNH | 81250 | Cercocebus agilis | Papionini | Male | EG | 24.1 | 5.6 | 6.5 | 39.4 | 7.9 | 5.5 | 37.1 | 7.0 | 6.5 | 35.8 | 5.4 | 5.1 | ? | ? | ? |
| AMNH | 52634 | Cercocebus agilis | Papionini | Male | EG | 24.1 | 6.8 | 7.9 | 40.2 | 8.7 | 6.3 | 38.9 | 7.3 | 7.0 | 38.0 | 6.7 | 5.6 | 36.3 | 8.1 | 5.2 |
| AMNH | 52596 | Lophocebus albigena johnstoni | Papionini | Female | EG | 20.1 | 6.0 | 6.1 | 36.9 | 7.9 | 5.7 | 37.6 | 7.1 | 6.3 | 37.8 | 6.5 | 5.2 | 34.5 | 7.5 | 5.3 |
| AMNH | 52603 | Lophocebus albigena johnstoni | Papionini | Male | EG | 24.1 | 7.0 | 7.6 | 44.0 | 8.3 | 6.1 | 43.4 | 8.1 | 7.2 | 41.8 | 8.1 | 5.7 | 39.8 | 6.6 | 6.9 |
| USNM | 573504 | Macaca fascicularis | Papionini | Male | EG | 19.9 | 5.7 | 6.0 | 33.3 | 7.3 | 6.1 | 30.9 | 6.7 | 6.3 | 29.9 | 6.5 | 4.7 | 27.3 | 4.9 | 5.2 |
| USNM | 305069 | Macaca nemestrina | Papionini | Female | EG | 23.5 | 5.7 | 7.3 | 39.9 | 8.2 | 6.0 | 38.6 | 7.4 | 6.3 | 37.3 | 7.0 | 5.7 | 34.8 | 4.5 | 5.1 |
| USNM | 49874 | Macaca nemestrina | Papionini | Male | EG | 27.3 | 7.7 | 8.4 | 45.4 | 10.3 | 7.3 | 44.2 | 9.1 | 8.4 | 42.8 | 8.4 | 6.6 | 41.4 | 7.4 | 7.5 |
| USNM | 49691 | Macaca nemestrina | Papionini | Male | EG | 29.1 | 7.5 | 7.6 | 47.7 | 9.3 | 6.6 | 46.7 | 8.8 | 7.8 | 45.3 | 7.8 | 6.3 | 45.8 | 6.9 | 7.4 |
| AMNH | 106564 | Macaca nemestrina | Papionini | Male | EG | 31.1 | 8.3 | 9.4 | 49.9 | 11.1 | 8.0 | 47.2 | 9.2 | 8.8 | 46.3 | 8.8 | 6.8 | 44.7 | 7.8 | 8.4 |
| USNM | 241163 | Macaca thibetana | Papionini | Male | EG | 23.8 | 7.8 | 7.3 | 42.2 | 9.8 | 7.6 | 40.3 | 8.1 | 8.2 | 39.3 | 7.7 | 6.6 | 37.8 | 7.7 | 8.7 |
| USNM | 258686 | Macaca thibetana | Papionini | Male | EG | 19.0 | 6.6 | 6.9 | 37.2 | 8.2 | 7.2 | 35.2 | 8.3 | 8.2 | 34.0 | 7.4 | 6.1 | 32.0 | 7.2 | 7.0 |
| USNM | 258649 | Macaca thibetana | Papionini | Male | EG | 20.8 | 7.6 | 7.6 | 40.6 | 8.6 | 7.6 | 38.3 | 8.5 | 8.4 | 37.6 | 8.4 | 6.5 | 35.5 | 8.1 | 8.5 |
| AMNH | 60160 | Macaca thibetana | Papionini | Male | EG | 22.0 | 8.1 | 8.1 | 38.3 | 9.4 | 7.8 | 38.8 | 8.5 | 8.2 | 38.2 | 8.3 | 6.8 | 37.1 | 8.2 | 7.8 |
| USNM | 258651 | Macaca thibetana | Papionini | Male | EG | 22.1 | 8.3 | 8.3 | 40.6 | 9.2 | 7.4 | 39.8 | 9.0 | 8.8 | 37.8 | 8.6 | 7.1 | 35.3 | 7.8 | 8.0 |
| USNM | 254800 | Macaca thibetana | Papionini | Male | EG | 22.6 | 6.9 | 7.4 | 39.3 | 8.9 | 7.3 | 37.6 | 8.1 | 8.4 | 35.9 | 7.6 | 7.1 | ? | ? | ? |
| AMNH | 89358 | Mandrillus sphinx | Papionini | Female | EG | 31.3 | 7.2 | 9.1 | 48.1 | 9.5 | 7.5 | 46.6 | 9.8 | 8.7 | 47.2 | 8.5 | 6.4 | 48.1 | 7.1 | 10.4 |
| KNM | OM 7230 | Papio hamadryas anubis | Papionini | Female | SF | 29.5 | 7.3 | 9.0 | 44.4 | 11.2 | 8.7 | 47.8 | 10.3 | 8.1 | 46.3 | 10.2 | 6.9 | 43.9 | 7.6 | 7.8 |
| USNM | 384228 | Papio hamadryas anubis | Papionini | Male | EG | 30.3 | 7.7 | 9.9 | 50.8 | 13.1 | 8.3 | 49.0 | 10.8 | 9.3 | 48.9 | 11.1 | 7.8 | 48.8 | 8.1 | 8.6 |
| USNM | 384234 | Papio hamadryas anubis | Papionini | Male | EG | 37.3 | 9.2 | 11.0 | 57.8 | 15.1 | 9.2 | 57.0 | 13.0 | 12.5 | 55.7 | 13.2 | 9.0 | 55.3 | 9.6 | 9.7 |
| USNM | 236976 | Papio hamadryas anubis | Papionini | Male | EG | 39.6 | 9.1 | 11.2 | 62.4 | 14.1 | 10.5 | 59.3 | 13.0 | 10.4 | 60.2 | 12.6 | 8.6 | 58.9 | 11.4 | 11.0 |
| KNM | OM 5179 | Papio hamadryas anubis | Papionini | Male | SF | 35.8 | 9.7 | 11.0 | 56.2 | 12.8 | 8.7 | 54.9 | 12.0 | 9.4 | 53.9 | 11.7 | 7.9 | 52.7 | 9.0 | 9.4 |
| USNM | 239743 | Papio hamadryas anubis | Papionini | Unknown | EG | 34.6 | 7.8 | 10.5 | 54.5 | 11.5 | 7.9 | 53.0 | 10.8 | 9.0 | 52.8 | 10.3 | 7.1 | 54.5 | 8.1 | 8.5 |
| HERC | 113 | Theropithecus gelada | Papionini | Female | SF | 28.1 | 6.2 | 7.9 | 37.4 | 8.8 | 6.9 | 38.4 | 7.7 | 7.9 | 37.0 | 7.7 | 5.8 | 37.3 | 6.1 | 7.1 |
| HERC | 110 | Theropithecus gelada | Papionini | Female | SF | 30.5 | 7.6 | 6.3 | 41.7 | 8.9 | 6.6 | 42.0 | 7.3 | 8.2 | 40.9 | 6.2 | 7.8 | 40.4 | 5.9 | 8.2 |
| NME | Monkey "F" 1 | Theropithecus gelada | Papionini | Female | SF | 30.4 | 6.3 | 7.6 | 40.4 | 9.3 | 6.8 | 39.0 | 9.0 | 8.2 | 39.4 | 8.7 | 7.0 | 38.1 | 6.4 | 7.6 |
| HERC | 109 | Theropithecus gelada | Papionini | Male | SF | 33.4 | 6.8 | 9.1 | 45.2 | 10.1 | 7.9 | 45.4 | 9.1 | 8.8 | 44.9 | 9.0 | 6.5 | 44.6 | 7.2 | 8.0 |
| HERC | 108 | Theropithecus gelada | Papionini | Male | SF | 35.0 | 7.0 | 9.2 | 47.4 | 10.3 | 7.8 | 46.6 | 9.6 | 8.8 | 45.1 | 8.7 | 7.1 | 45.6 | 7.2 | 8.4 |
| AMNH | 201008 | Theropithecus gelada | Papionini | Male | EG | 37.0 | 7.2 | 8.5 | 48.0 | 10.6 | 8.3 | 47.1 | 9.1 | 9.5 | 47.1 | 9.2 | 8.2 | 46.0 | 10.6 | 7.1 |
| MNHN | ABB.86 | Mesopithcus pentelicus | Fossil Colobine | Female | Z91 | 18.0 | 5.0 | 5.7 | 30.2 | 7.2 | 4.5 | 30.3 | 6.0 | 5.5 | 27.8 | 6.0 | 4.6 | 26.2 | 5.3 | 4.8 |
| MNHN | ABB.91 | Mesopithcus pentelicus | Fossil Colobine | Female | Z91 | 18.0 | ? | ? | ? | ? | ? | 32.0 | ? | 5.8 | 29.0 | ? | 4.6 | ? | ? | ? |
| MNHN | ABB.92 | Mesopithcus pentelicus | Fossil Colobine | Male? | Z91 | 18.5 | ? | ? | 31.5 | ? | ? | 33.0 | ? | 6.7 | 31.5 | ? | ? | 28.5 | ? | ? |
| MNHN | ABB.88 | Mesopithcus pentelicus | Fossil Colobine | Male? | Z91 | ? | ? | ? | ? | ? | ? | 34.3 | 7.5 | 6.2 | 32.6 | 7.2 | 5.2 | ? | ? | ? |
| KNM | KNM-NA 47916 | Microcolobus tugenensis | Fossil Colobine | Unknown | N10 | ? | 4.9 | 5.7 | ? | ? | ? | ? | ? | ? | 30.4 | ? | ? | ? | ? | ? |
| KNM | KNM-ER 4420 | Cercopithecoides willliamsi | FossilAfrica | Male | CG | ? | 8.3 | 7.7 | ? | 12.9 | 9.6 | 58.5 | 13.3 | 9.2 | 55.5 | 11.8 | 9.8 | 55.4 | 9.9 | 8.9 |

Key to observers: SF, CG, EG, KP authors; Z91 Zapfe (1991); N10 Nakatsukasa et al. (2010)
