# Supplementary material for: The Hand of Cercopithecoides williamsi (Mammalia, Primates): Earliest Evidence for Thumb Reduction among Colobine Monkeys
Source: PLoS One. 2015 May 20;10(5):e0125030. doi: 10.1371/journal.pone.0125030 (PMC4439063; doi:10.1371/journal.pone.0125030)
Supplement: S2 Table — (DOCX) [file pone.0125030.s002.docx]

**Table S2.** Loadings from principal components analysis.
